# An information-theoretic analysis of resting-state versus task fMRI

**DOI:** 10.1162/netn_a_00302

**Published:** 2023-06-30

**Authors:** Julia Tuominen, Karsten Specht, Liucija Vaisvilaite, Peter Zeidman

**Affiliations:** Department of Biological and Medical Psychology, University of Bergen, Bergen, Norway; Department of Global Public Health and Primary Care, University of Bergen, Bergen, Norway; Mohn Medical Imaging and Visualization Centre, Haukeland University Hospital, Bergen, Norway; Department of Education, The Arctic University of Norway UiT, Tromsø, Norway; Wellcome Centre for Human Neuroimaging, UCL Institute of Neurology, University College London, London, United Kingdom

**Keywords:** Bayesian data comparison, Effective connectivity, Resting-state, Task fMRI, Data quality

## Abstract

Resting-state fMRI is an increasingly popular alternative to task-based fMRI. However, a formal quantification of the amount of information provided by resting-state fMRI as opposed to active task conditions about neural responses is lacking. We conducted a systematic comparison of the quality of inferences derived from a resting-state and a task fMRI paradigm by means of Bayesian Data Comparison. In this framework, data quality is formally quantified in information-theoretic terms as the precision and amount of information provided by the data on the parameters of interest. Parameters of effective connectivity, estimated from the cross-spectral densities of resting-state- and task time series by means of dynamic causal modelling (DCM), were subjected to the analysis. Data from 50 individuals undergoing resting-state and a Theory-of-Mind task were compared, both datasets provided by the Human Connectome Project. A threshold of very strong evidence was reached in favour of the Theory-of-Mind task (>10 bits or natural units) regarding information gain, which could be attributed to the active task condition eliciting stronger effective connectivity. Extending these analyses to other tasks and cognitive systems will reveal whether the superior informative value of task-based fMRI observed here is case specific or a more general trend.

## Author Summary

The ongoing replication crisis in neuroscience and the concurrent “paradigm shift” from task-based to resting-state fMRI raises a question about the relative quality of the data obtained from these imaging paradigms. We compared parameters of intrinsic effective connectivity estimated from resting-state and Theory-of-Mind datasets. The much weaker connectivity and consequent lower information gain of the resting condition was notable as the network was specified based on connectivity patterns observed under rest and consisted of regions associated with the default mode network, which is characterized by being active during rest. These results support the assumption that the resting connectivity of the default mode network may reflect physiological rather than neural processes, and that the neural system in question better lends itself to investigation under an active task condition.

## INTRODUCTION

In functional magnetic resonance imaging (fMRI) research, the shift from functional localization to functional and effective connectivity as the primary object of investigation has been naturally accompanied by a so-called “paradigm shift” in the design of imaging protocols (Raichle, 2009). Resting-state fMRI (rs-fMRI) has become an attractive alternative to task-based fMRI (t-fMRI) due to ease and efficiency of acquisition (Dubois & Adolphs, 2016; Leuthardt et al., 2015), and because the intrinsic functional connectivity of the brain can be investigated without a known timeline of experimental events (Cole et al., 2014). Yet, research questions posed in the context of t-fMRI are not restricted to those pertaining to functional localization. Both functional and effective connectivity can be and are being studied under different task conditions, revealing temporally coherent networks congruent with those observed in rest (Calhoun et al., 2008; Cole et al., 2014; Kieliba et al., 2019; Smith et al., 2009). Furthermore, clinically relevant individual differences have been demonstrated to be preserved across rest and different active tasks (Mwansisya et al., 2017; Schurz et al., 2015). Followingly, the (dys)function of a given neural system can be investigated under both imaging paradigms, which makes inquiries into the relative quality of the obtained data highly relevant.

Studies that have compared the performance of t-fMRI to rs-fMRI data suggest the superiority of t-fMRI in measures such as predictive accuracy and reliability (Finn et al., 2017; Frässle & Stephan, 2022; Gaut et al., 2019; Greene et al., 2018; Kristo et al., 2014; McCormick et al., 2022; Noble et al., 2019; Rosazza et al., 2014; Specht et al., 2003; Wang et al., 2017; Weber et al., 2013; Xie et al., 2018; Yoo et al., 2018). Performance in such measures is of increasing importance given the growing interest in applying fMRI in the identification of disease biomarkers and in individualized treatment approaches (Brooks & Vizueta, 2014; Carter et al., 2008; McDermott et al., 2018). In clinical applications the measurement of activation strength, such as the strength of between-region coupling, becomes particularly important, which introduces additional demands on data quality (Specht, 2020).

In this study the question about data quality was formulated as to whether rs- or t-fMRI data provide more information about neural responses, the relative precision with which we can infer the strength of individual connection parameters, as well as the ability to distinguish between different neural network architectures. These can be formally assessed with an information-theoretic approach in terms of reduction in entropy. The present question could therefore be tackled using Bayesian data comparison (BDC), an analysis framework introduced by Zeidman et al. (2019c) that draws on Bayesian statistics to measure movement from prior to posterior distributions afforded by the data. Neural responses, defined as the rate of change in the activity of neural populations in units of s^−1^ (Hz), cannot be directly observed using BOLD fMRI but can only be inferred under a parameterized generative model. Therefore, BDC was here applied in the context of dynamic causal modelling (DCM) to obtain parameters of effective connectivity, which is simply the contribution of one population’s neural response towards another’s. Our research question can formally be stated as: how much more information do we have about the unknown parameters governing neural responses after observing each kind of data.

The data quality indices obtained from BDC have been demonstrated to be positively correlated with signal-to-noise ratio (SNR), a metric commonly used to quantify the quality of fMRI data (Bennett & Miller, 2010; Welvaert & Rosseel, 2013; Zeidman et al., 2019c). However, unlike SNR that is insensitive to the feature of interest in connectivity studies, the present indices measure data quality in relation to the studied connectivity parameters. Thus, BDC enables an analysis of data quality beyond that indicated by SNR or the relative amount of within- and between-individual variability, which is typically quantified in studies of reliability and predictive accuracy.

We envisage that comparing the performance of several datasets in the context of a particular network model would be especially useful in clinical settings, where the focus shifts from identifying the affected neural system to optimizing the imaging paradigm under which to best measure its function. We hypothesized that perturbations of a neural network by external stimuli, which evoke cognitive processes supported by that network, will facilitate the estimation of effective connectivity parameters and lead to better performance in terms of the above-mentioned quality indices. We used data from the Human Connectome Project (Van Essen et al., 2013), which is particularly suitable for the present purpose due to being acquired under both resting-state and different task conditions using the same acquisition protocol and the same set of subjects, in addition to coming from a quality-assured, open data source.

## MATERIALS AND METHODS

### The Data

We employed the minimally preprocessed data provided by the Human Connectome Project (HCP). The sample consisted of 50 subjects in total, 23 males and 27 females between the ages 22 and 35, selected from the predefined subset of 100 unrelated subjects of the Young Adult dataset. Informed consent was obtained from the subjects both upon initial screening and at the beginning of the scanning session (Van Essen et al., 2013). The present study did not necessitate the use of data from siblings or twins, or biological data from HCP Restricted Data, which are considered more sensitive and are available only through a separate application process (Van Essen et al., 2013). Furthermore, the results will be reported only at the group level, such that any risk of identification should be minimal. The authors of the present study have agreed to the Open Access Data Use Terms of HCP, and the Norwegian Regional Committees for Medical and Health Research Ethics (REK) approved the use of HCP data in the project “When Default is not Default,” of which the present study is part of (REK West: 31972).

HCP provides data on the same subjects undergoing two rs-fMRI sessions and seven t-fMRI sessions. The different tasks have been demonstrated to recruit a wide range of well-characterized neural systems efficiently and reliably (Barch et al., 2013). For the current analysis, we selected a social cognition task adapted from the ones developed by Castelli et al. (2000) and Wheatley et al. (2007). It consists of social animation stimuli in the form of video clips of geometrical objects moving either randomly or in a biologically meaningful pattern, which were rated online by the participants based on whether they were perceived to involve social interaction. The cognitive processes deliberately evoked by this type of task, collectively known as the Theory-of-Mind (ToM), are suggested to occur spontaneously during rest. Similar to other tasks engaging such “self-referential” processes, it has been observed to activate parts of the default mode network (DMN) (Andreasen et al., 1995; Mars et al., 2012; Schilbach et al., 2008; Spreng & Grady, 2010; Spreng et al., 2009). It was therefore particularly interesting to compare data from this task, hereafter referred to as the ToM task, to data from a rs-fMRI session for the same group of subjects.

In HCP, fast sampling with TR of 720 ms and TE of 33 ms is used (Glasser et al., 2013). A detailed account of the image acquisition protocol and the HCP minimal preprocessing pipeline can be found in Van Essen et al. (2012) and Glasser et al. (2013). Due to the specific image acquisition protocol employed in HCP there were two runs of each imaging condition, scanned with reversed phase encoding directions (Van Essen et al., 2013). For simplicity, in our analysis we included only data from Run 2 of the ToM task scanned in the left-right direction, consisting of three clips of social interaction and two clips of random movement. Each run started with a countdown of 8 s, and the duration of each animation clip was 20 s, followed by 3 s for a behavioural response and a 15-s fixation block. One complete run therefore lasted for 3 min and 27 s. Data from a resting-state (RS) session carried out on the same day was utilized to avoid possible differences in the two datasets arising from factors fluctuating on a daily basis. For the sake of consistency, we utilized the preprocessed datasets in which ICA-based denoising has not been applied, as this option is available only for rs-fMRI data. Again, data acquired with the left-right scanning direction was employed, which was always the first run. The duration of the run was 14 min 33 s. In the RS condition the participants were requested to lie with their eyes open and fixated on a white cross on a dark background, to think of nothing particular, and not to fall asleep (Smith et al., 2013). The use of concatenated images across the opposite scanning directions was assessed to potentially increase overall data quality but to greatly complicate the preparatory data processing without changing the relative quality of the two datasets. As our main interest was in the relative and not in the absolute data quality, only the images acquired with one scanning direction were included in the analysis.

### Preparatory Analyses

The minimally preprocessed data were smoothed with an 8-mm Gaussian kernel, using SPM-12 (v7771) in MATLAB 2019a. Thereafter, a standard univariate general linear model (GLM) was conducted on the ToM data. In the first-level GLM analysis a design matrix was specified, which included the countdown, block, response, and fixation times specified above. The default options of microtime resolution and onset of 16 and 18, high-pass filter 128 s and canonical hemodynamic response function (HRF) convolution model were applied, and the 12 movement parameters (translation, rotation, and their derivatives) were included as covariates in the design matrix. Countdown, fixation, and response times were included as regressors in order to obtain a map of activation specific to viewing social stimuli. A contrast between blocks of socially meaningful movement and blocks of random movement (Social > Random) was defined. In the second-level group analysis a one-sample *t* test was calculated for this contrast to identify regions specifically and significantly responsive to social interaction. The results were exported as a binary mask to be used at the subsequent stages of the analysis.

An independent component analysis (ICA) was conducted on the RS dataset with the purpose of identifying a component that best corresponds to the activation map obtained from the preceding GLM analysis, thereby thought to reflect the intrinsic connectivity of regions associated with ToM processes. The RS images were also smoothed in SPM-12 with an 8-mm smoothing kernel before importing the files to the GIFT-toolbox v3.0b in MATLAB 2019a, where the ICA was performed. The number of 42 components, advocated in some sources (Kiviniemi et al., 2009), was considered high enough not to result in wide, functionally heterogeneous networks but neither in overly circumscribed within-region networks. The default algorithm Infomax was applied. The stability of the derived components was analysed with ICASSO that repeated the analysis 10 times. The spatial configurations of the components were individually reconstructed and sorted according to their spatial overlap with the binary mask extracted from the GLM analysis of ToM data. This allowed us to identify a RS independent component that best overlapped with the ToM activation map. The reconstructed maps of this component from each subject were imported to SPM-12, where a one-sample *t* test was conducted to obtain a group-level spatial map of significant clusters. The coordinates of five most significant clusters were used as nodes in the following DCM analysis. These five clusters are listed in Table 1.

**Table 1.** Coordinates of the DCM nodes extracted from the best matching independent component

Label	MNI^1^			T-value
	x	y	z	
R angular gyrus	56	−50	20	30.94
R superior temporal sulcus	54	−46	6	25.24
R/L precuneus	4	−54	60	13.23
R temporal pole	50	−6	−18	12.44
R fusiform gyrus	44	−50	−20	7.01

*Note*. R = Right, L = Left. FWE correction at *p* < 0.05 and cluster level 20 were used.

^1^ Montreal Neurological Institute brain coordinates.

### Main Analysis

#### Time series extraction.

While the time courses of the ToM task were directly extracted from the preparatory individual first-level GLM analysis, the RS data needed some further processing. The RS time series was reduced from the original scanning length of 14 min 33 s to the same length as the ToM task, that is 3 min and 27 s. This ensured a more formal comparison of data quality not influenced by the accumulation of signal across time, which is known to increase SNR. As an additional analysis, we compared ToM data also to the full-length RS data, as this is more consistent with the typical application of rs-fMRI. First, a dummy GLM was set up to extract time series from the RS data, followed by another GLM in which the 12 movement regressors and signals from white matter [0, −24, −33] and cerebrospinal fluid [0, −40, −5] were used to regress out further noise related to motion, scanner, and physiological processes (Weissenbacher et al., 2009). The same high-pass filter as for the ToM data analysis was applied. Time series for the five nodes were extracted by centring spherical regions with a radius of 8 mm on their coordinates, from which the first principal eigenvariate of all voxels within the sphere, centred on the peak voxel, summarized the time series of a given node. The time series were mean corrected. Motion correction and time series extraction proceeded in a similar manner for ToM data.

#### Dynamic causal modelling.

The measures of data quality applied here depend on both the efficiency of the selected tasks for inducing effective connectivity among the regions of interest, and the efficiency of the model for inferring the presence of those effects. As our focus was comparing tasks, we kept the model as consistent as possible across datasets by using the same forward model with the same regions of interest and connectivity architectures.

We employed DCM for cross-spectral densities (csd-DCM) in SPM-12 to invert a neural network model consisting of the nodes identified at the preparatory stage of the analysis (Razi et al., 2015). This was done separately for RS and ToM data, which produced subject-specific estimates of intrinsic effective connectivity. DCM for CSD was used due to it being applicable to both t-fMRI and rs-fMRI data (Friston et al., 2014), although this version of DCM does not allow the testing of condition-specific modulations on effective connectivity (i.e., no B matrix). However, this is not relevant for the present study, where the t-fMRI data only included a single experimental factor (social vs. random movement). Therefore, only the blocks of social stimuli were included as driving input through the fusiform gyrus (C matrix) when modelling connectivity during the ToM task. The quality indices were based on the invariant connectivity (A matrix) of the respective dataset.

We allowed all connections between the regions to be informed by the data. In the within-subject (DCM) and between-subject (parametric empirical Bayes, PEB) models, priors on parameters were left at their default values, as supplied with the SPM12 software. Priors at the within-subject subject level are detailed in table 3 of Zeidman et al. (2019a) and priors at the between-subject level are detailed in appendices 1 and 2 of Zeidman et al. (2019b). The most important parameters in the DCM neural model for the analyses presented were those forming the region-by-region effective connectivity matrix (matrix A). To briefly reprise, this was multivariate normal prior, where the connection from region *j* to region *i* had the probability density *A*_*ij*_ ∼ *N*(0, 1/64). This is referred to as a shrinkage prior, because in the absence of evidence to the contrary, it assumes no connectivity (0 Hz) among regions.

#### Bayesian data comparison.

The subject-specific effective connectivity parameters were subjected to the BDC analysis pipeline (spm_dcm_bdc.m, revision 7495) in SPM-12. Whereas standard statistics based on likelihood ratios are used to compare the evidence for different models fitted to the same data (e.g., *F* tests, Bayes factors), these statistics cannot be used to compare models fitted to different data. The BDC procedure works around this by evaluating which dataset affords the greatest precision or confidence about the model parameters and the models themselves. Ideally, one would follow standard statistical procedure for Bayesian hypothesis testing, which is to evaluate the log evidence ln *P*(*y*|*m*_*i*_) for each model of interest *m*_*i*_, and then compare them by computing the log Bayes factor. For two models, the log Bayes factor is simply the difference in log evidences, ln *P*(*y*|*m*_1_) − ln *P*(*y*|*m*_2_). However, this assumes that the data *y* are the same for each model, which precludes the use of the Bayes factor for comparing models fitted to different datasets. To address this, the log evidence (and its free energy approximation used here) can be decomposed into the difference between the model’s accuracy and complexity:

$$\ln P\left(y|m_{i}\right)=\underset{\text{accuracy}}{\underbrace{{\langle \ln P\left(y|\theta ,m_{i}\right)\rangle }_{p\left(\theta |y,m_{i}\right)}}}-\underset{\text{complexity}}{\underbrace{KL\left[P\left(\theta |y,m\right)\|P\left(\theta |m\right)\right]}}$$

In words, the accuracy is the expected log likelihood (the probability of observing the data under the model after estimating the parameters), which would not be meaningful to compare across datasets. The complexity, also called the relative entropy, describes how far the parameters have diverged from their prior expectations. This is quantified by the Kullback–Leibler (KL) divergence, also called the relative entropy, which is a measure of difference between two probability distributions (Joyce, 2011):

$${\text{D}}_{\text{KL}}\left[P\left(\beta^{\left(\text{i}\right)}|Y^{\left(\text{i}\right)}\right)∥P\left(\beta^{\left(\text{i}\right)}\right)\right]$$

This quality index considers the prior and posterior expected values of the parameters ($\mu_{0}^{\left(i\right)}$, *μ*^(*i*)^) and the covariance matrices ($\Sigma_{0}^{\left(i\right)}$, Σ^(*i*)^), which determines the effective number of independent parameters that the data can support (Zeidman et al., 2019c). This measures how much information has been gained after observing the data and has natural units (nats), which enable a convenient comparison between datasets. For this reason, it is used in BDC as the basis for comparing how much has been learnt from each dataset. A difference between 1.1 and 3 nats can be described as positive evidence in favour of one dataset over another, a difference between 3 and 5 nats as strong evidence, and differences beyond that indicate very strong evidence (akin to a Bayes Factor; Kass & Raftery, 1995).

The central steps of BDC are as follows. First, connection parameters are estimated for each subject and are then optimized after obtaining the average connectivity of the group (empirical Bayes). In more detail, for each subject *s* and dataset *i*, a generative model of fMRI data is specified:

$$\text{vec}\left(Y^{\left(i,s\right)}\right)=f\left(\theta^{\left(i,s\right)}\right)+\epsilon^{\left(i,s\right)}$$

where *Y* is a matrix of fMRI timeseries data, the vec(·) operator converts a matrix to a vector, *f* is a model (here, an fMRI DCM), *θ* is a vector of connection parameters and *ϵ* are the residuals. Model fitting is then performed for each subject, to obtain a posterior probability distribution over the parameters, *P*(*θ*^(*i*,*s*)^|*Y*^(*i*,*s*)^), as well as score for the quality of the model, the log evidence, which is approximated by the free energy *F*^(*i*,*s*)^ ≈ ln *P*(*Y*^(*i*,*s*)^). All subjects’ models are then reestimated, using the group-average connection parameters (across all subjects and both datasets) as priors. This reestimation procedure can “rescue” any subjects whose parameters have fallen into different local optima.

Next, the optimized subject-specific connectivity parameters are summarized at the group level, separately for each dataset, using a hierarchical PEB model. This Bayesian scheme provides an estimated probability distribution over the group-average connectivity parameters as well as a score for the quality of the complete hierarchical model—the free energy—for each dataset. These two outputs—the group-level parameters and the free energy—form the basis for the comparisons that follow. More formally, for each dataset *i* = 〈1, 2〉 and subject *s*, we have a hierarchical model:

$$\left(\text{Level}\;2\right)\;\theta^{\left(i\right)}=X\beta^{\left(i\right)}+E^{\left(i\right)}$$

$$\left(\text{Level}\;1\right)\;\text{vec}\left(Y^{\left(i,s\right)}\right)=f\left(\theta^{\left(i,s\right)}\right)+\epsilon^{\left(i,s\right)}$$

where *θ*^(*i*)^ is a vector of all subjects’ connection parameters, the design matrix *X* encodes any between-subjects effects, *β* are the parameters encoding the group-average connectivity and the effects of any covariates on the connections, and *E* is the unexplained between-subjects variability. Constraints are imposed on the group-level parameters through a prior probability distribution, *P*(*β*^(*i*)^) = *N*($\mu_{0}^{\left(i\right)}$, $\Sigma_{0}^{\left(i\right)}$). Estimating the model supplies a posterior probability distribution over the group-level connection parameters, informed by all subjects, which is multivariate normal *P*(*β*^(*i*)^|*Y*^(*i*)^) = *N*(*μ*^(*i*)^, Σ^(*i*)^), as well as the overall free energy of the hierarchical model *F*^(*i*)^ ≈ ln *P*(*Y*^(*i*)^).

Three statistics are then computed in order to compare the hierarchical models fitted to the different datasets, referred to as (1) parameter certainty, (2) information gain over parameters and (3) information gain over models.

The *parameter certainty* is the confidence with which the connection parameters have been estimated at the group level. It is a function of the posterior covariance matrix Σ^(*i*)^, which encodes the uncertainty or variance of each parameter on its leading diagonal, and the covariance among parameters on the off-diagonal entries. The covariance determines the extent to which parameters can be distinguished from each other, which is important when testing hypotheses that consist of several parameters being estimated simultaneously. The parameter certainty *S*^(*i*)^ is defined as the negative entropy of the covariance matrix:

$$S^{\left(i\right)}=-0.5\ln\left|2\pi e\Sigma^{\left(i\right)}\right|$$

The dataset with highest negative entropy affords greater reduction in uncertainty, measured in nats.

The *information gain over parameters* scores the reduction in uncertainty after seeing the data relative to the uncertainty before seeing the data. It quantifies how far the parameters have changed from prior beliefs *P*(*β*^(*i*)^) to the posterior beliefs *P*(*β*^(*i*)^|*Y*^(*i*)^), that is, the relative entropy as explained above.

Finally, *information gain over models* indicates the ability to discriminate between models and to identify the optimal one (Zeidman et al., 2019c). In BDC, a model space consisting of equally plausible but difficult to distinguish models is formed, by switching on or off particular connections in the neural network. In the current case, only the A matrix from the DCM models served as input for the BDC procedure. The models are assigned equal prior probabilities, forming a discrete prior probability distribution over models. The posterior probability of each model is calculated, and the KL-divergence between the prior and posterior probability distributions is computed (Zeidman et al., 2019c). The less uniformly the posterior probability is distributed among the models (i.e., the more that some models are favoured over others), the higher KL-divergence, indicating better discrimination among models and greater information gain over the model structure (see Formula S1). An analytic approach for rapidly approximating the posteriors and model evidence called Bayesian model reduction (BMR) is used to reduce computation time (Friston et al., 2016).

In summary, the BDC procedure involves fitting models (DCMs) to each subject’s fMRI data, then taking the model parameters from all subjects up to the group level and fitting a GLM for each dataset under consideration. The parameters of each GLM quantifies the group-average connectivity, which are used to compute the statistics described above.

An additional step originally proposed in the BDC pipeline by Zeidman et al. (2019c) was not applied here. They used BMR to prune away any redundant parameters that do not contribute to model evidence at the group-level GLM. It results in one parsimonious model informed equally by all datasets, the parameters of which are used to reestimate individual-level connections. In this study, BDC was conducted on a fully connected model by turning off the BMR function. This was done because the network structure may differ under rs- and t-fMRI and fitting the data to a model that represents a compromise between the two imaging conditions might have unpredictable effects on the rest of the analysis.

## RESULTS

**Figure 1.**
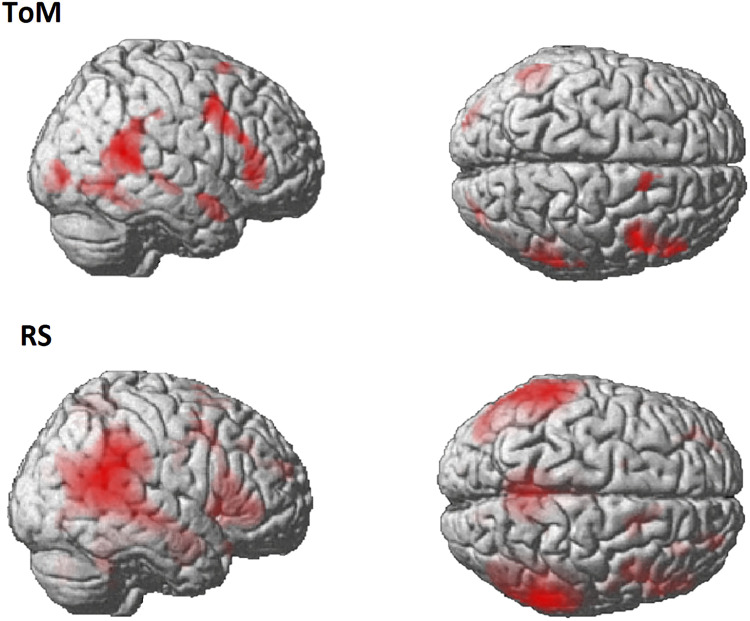
Activation map for the contrast Social > Random in the ToM task and the independent RS component that best overlapped with that map, both displayed with *p*(FWE) < 0.05 and cluster level 20.

**Figure 2.**
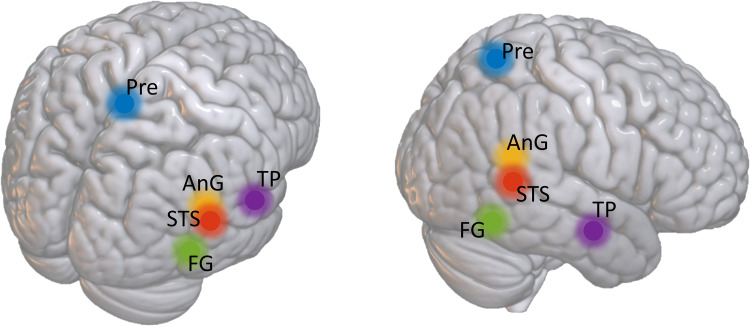
Approximate locations of the five nodes included in the csd-DCM.

The comparison between the ToM activation map obtained with the GLM and the 42 RS independent components obtained with the ICA identified one component that covered a substantial part of the ToM activation map. A spatial map of this component and the ToM activation map are displayed in Figure 1. The activation peaks of the ToM task can be found in the Supporting Information (Table S1). The five most significant regions identified with the group-level one-sample *t* test on the RS component were located in the right superior temporal sulcus (rSTS), (bilateral) precuneus, right angular gyrus (rAnG), right temporal pole (rTP), and right fusiform gyrus (rFG). The MNI coordinates of their cluster peaks are summarized in Table 1 and approximate locations are displayed in Figure 2.

**Figure 3.**
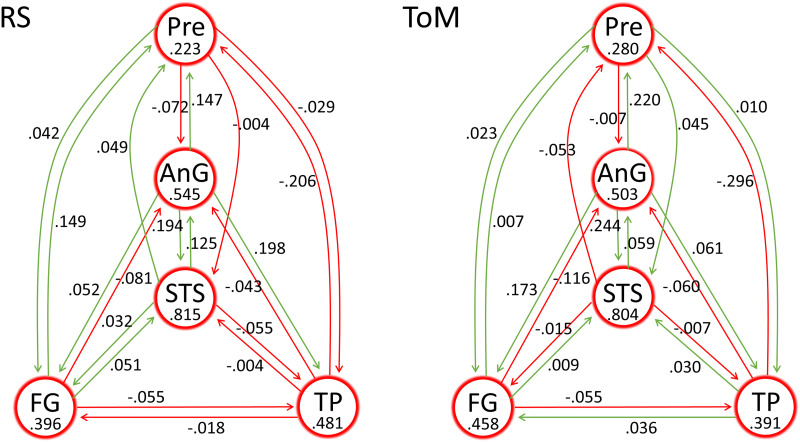
Effective connectivity during RS and ToM. The values for between-region connections are in units of Hz whereas self-connections are unitless log-scaling values. Green and red colours represent excitatory and inhibitory connectivity, respectively. For the self-connections, positive values indicate more self-inhibition than the default value of −0.5 Hz, indicated with red circles.

The patterns of effective connectivity among the five nodes during the ToM task and during RS, directly after model inversion conducted independently on the two datasets, are displayed in Figure 3. Six out of 20 between-region connections displayed reversed direction of activity in terms of excitation and inhibition across the datasets. Activity within all five regions had stronger self-inhibition than the default value (−0.5 Hz) in both datasets.

**Figure 4.**
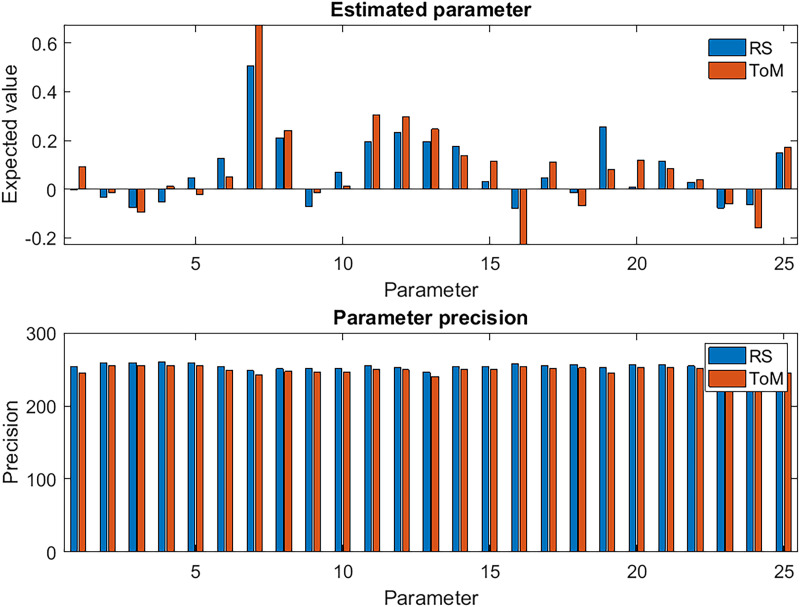
Estimated connectivity parameters. The parameters form a multivariate normal distribution with expected values illustrated in the top plot and precisions illustrated in the bottom plot. The parameters are ordered according to outgoing connections from precuneus (bars 1–5), STS (bars 6–10), AnG (bars 11–15), TP (bars 15–19), and FG (bars 20–25). The parameters for between-region connections are rate constants with units of Hz (s^−1^), whereas the self-connections are unitless log-scaling parameters, which control the level of inhibition in each region. Precision is the reciprocal of variance; therefore, the units are the reciprocal of the (squared) units of the parameters.

The connectivity parameters, after being reestimated with priors based on both datasets, are displayed in the upper graph of Figure 4. Although the sign and relative amplitude of the parameters is generally consistent between the two datasets, the majority of the connections in the ToM condition moved further from the default value of zero compared to RS, which indicates stronger connections in the ToM condition.

The parameter-specific precisions across the 25 within- and between-region connections, that is, our confidence about the connection strengths after seeing the data, are displayed in the lower plot of Figure 4. These precisions are defined as the inverse of the posterior variance diag(Σ^(*i*)^)^−1^. It is apparent that the precisions were consistent across connections and were slightly higher in the RS condition than the ToM condition. The information-theoretic analyses that follow quantify whether these differences across datasets were nontrivial.

**Figure 5.**
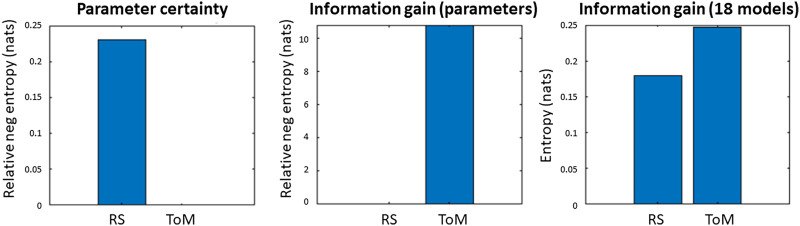
Parameter certainty and information gain over parameters and models in units of nats. The first two indices, parameter certainty and information gain over parameters, are negative entropies in units of nats, which are presented relative to the worst performing dataset (by subtracting the negative entropy of the best performing dataset from that of the worst performing dataset). The third index displays the amount of information gained with model comparison, separately for both datasets. Note the difference in scales—there was little difference in the parameter certainty (left) or information gain over models (right), but there was a large difference in the information gain over parameters (middle).

We summarised the estimated precision of the parameters across connections in terms of the *parameter certainty*, that is, the negative entropy, which also accounts for covariance between the parameters. This was 0.23 nats in favour of the RS dataset (left panel of Figure 5). This difference is trivially small and not large enough to count as positive evidence towards either condition. Thus, both conditions provided a similar level of confidence about the estimated values of the parameters.

The *information gain over parameters*, that is, the relative entropy from the priors to the posteriors, quantifies how much has been learnt by performing the experiment in units of nats. The difference in information gain between ToM and RS was above 10 nats and qualifies as very strong evidence in favour of t-fMRI (middle panel of Figure 5). Repeating the comparison using the full-length RS dataset did not noticeably change the relative information gain, which remained above 10 nats in favour of ToM (Figure S1). There was even a small decrease in the relative parameter certainty that was previously slightly more in favour of RS.

**Figure 6.**
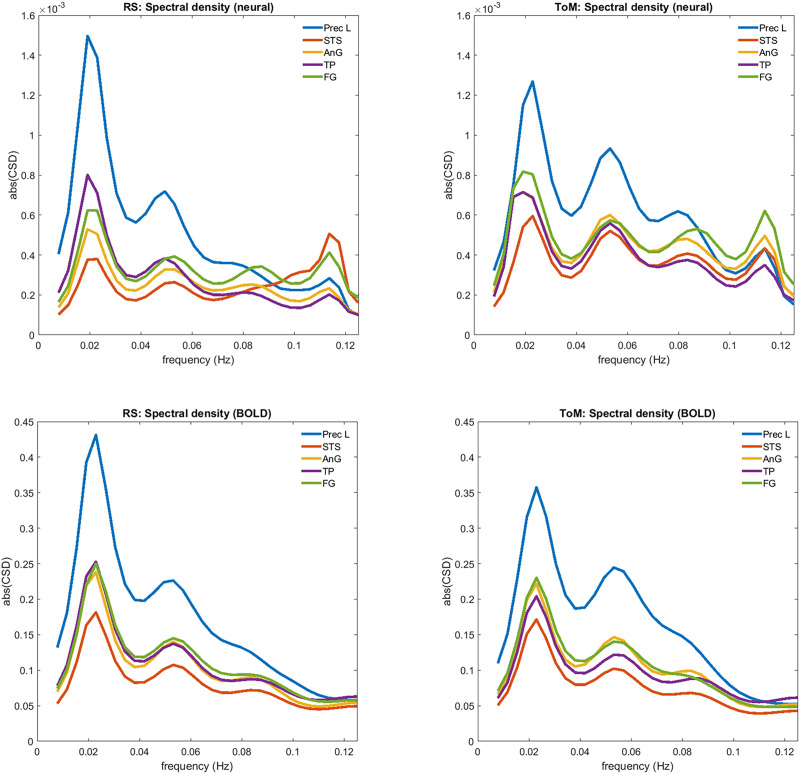
The subject-averaged power spectrum of each region corresponding to the estimated neural parameters (above) and the observed BOLD response (below) during RS and ToM. The numbers from DCM.Hs were averaged and plotted against DCM.Hz. The *y*-axis corresponds to power and *x*-axis to frequency.

Information gain depends on both the estimated strength of the connections and the precision of these estimates. As the precisions did not differ meaningfully between the two datasets (as quantified by the *parameter certainty* measure), the higher information gain connected to the ToM data was primarily driven by the stronger connection strengths. This is also evinced by the subject-averaged power spectrum of the estimated neural parameters associated with the datasets (Figure 6), which reveal higher amplitudes across different frequency bands and across most of the brain regions during the ToM task. More specifically, AnG and fusiform gyrus (FG) display higher amplitudes in the ToM condition across all frequency bands, whereas precuneus and temporal pole (TP) display lower amplitudes only at the lowest frequency and superior temporal sulcus (STS) at the highest frequency. The power spectrum of the observed BOLD response did not differ much between the datasets, except for the lowest frequency that showed higher amplitudes in most of the regions in the RS dataset.

The right panel of Figure 5 displays the information gained by comparing several equally plausible model structures. This analysis assessed whether ToM or RS data could best discriminate 18 difficult to disambiguate connectivity models. The information gain over model structure was 0.18 nats for RS and 0.25 nats for ToM. Both the amount of information gained after model comparison and the difference between the datasets in this index were negligible and do not count as evidence in favour of one or the other dataset.

## DISCUSSION

The relative merits of using an active task versus a passive task (rest) for eliciting measurable neural responses was assessed by conducting a systematic comparison of t-fMRI and rs-fMRI data, in information-theoretic terms. A social cognitive task evoking ToM processes served as the t-fMRI condition, which was chosen on the grounds of having previously demonstrated to activate brain regions attributed to the DMN (Andreasen et al., 1995; Mars et al., 2012; Schilbach et al., 2008; Spreng & Grady, 2010; Spreng et al., 2009). Parameters of effective connectivity derived by means of csd-DCM were subjected to the analysis.

Activation peaks of a group-level GLM across each individual’s reconstructed RS component were defined as DCM regions or nodes. The RS component was derived by means of ICA and selected based on its overlap with the ToM activation map. The nodes were located in rSTS, bilateral precuneus, rAnG, rTP, and rFG, each of which have been attributed to the DMN in earlier research (Greicius et al., 2003; Mars et al., 2012; Uddin et al., 2009). None of the core hubs of DMN in the middle frontal and cingulate regions were found significantly active in the current ToM task. To specifically target these regions, other ToM tasks such as false belief, trait judgement, autobiographical memory, or mind-in-the-eyes tasks could be considered (Andrews-Hanna et al., 2014; Schurz et al., 2014; Spreng et al., 2009).

Results from BDC show that the ToM task condition contributed significantly more information about the effective connectivity of the network model investigated here, compared to RS. The difference in information gain between the two datasets can be described as very strong evidence in favour of t-fMRI. Information gain depends on how far the parameters have moved from their prior expectation (zero) after seeing the data, as well as the confidence with which they could be estimated (their precision). In this case, the precisions of individual parameters were similar between the two datasets, but most of the connections were notably stronger in the ToM condition. This means that the higher information gain in the ToM task was primarily due to the stronger effective connectivity that it elicited compared to RS. Our results therefore demonstrate that the ToM task elicited stronger and more readily detectable effective connectivity than RS among regions associated with DMN. Connectivity strength has also been found to positively correlate with test-retest reliability in effective and functional connectivity measures (Frässle & Stephan, 2022; Noble et al., 2019).

A useful feature of DCM is that it discriminates between neural and haemodynamic components by modelling both separately to generate the observed signal, which enables the inference of connectivity parameters controlling hidden or latent neural activity (Friston et al., 2003, 2014). As the fMRI signal is an indirect measure of neural activity dependent on the level of blood oxygenation (Buxton, 2013), there are concerns that some patterns of connectivity are physiological rather than neural in origin (Ekstrom, 2010; Kelly et al., 2012; Lurie et al., 2020). Discerning the neural signal from physiological and background noise is more challenging in rs-fMRI compared to t-fMRI due to the lack of a control condition and a reference point provided by a time line of brain activity. Also, the slowly fluctuating neural activity measured in rs-fMRI may occupy the same frequency bands as noise (Birn et al., 2008; Dubois & Adolphs, 2016; Liu, 2016; Reid et al., 2019), and a considerable amount of rs-fMRI signal reliability and variability can be attributed to different noise sources (Almgren et al., 2020; Birn et al., 2014; Sjuls & Specht, 2022; Vaisvilaite et al., 2022; Wise et al., 2004; Yang et al., 2015). Consequently, the neural basis of the rs-fMRI signal and its relation to cognition and behaviour are a matter of uncertainty and there is a risk of confounding by physiological variables.

The results of the present analysis lend further support to this possibility, given the much lower information gain connected to rs-fMRI relative to t-fMRI. This is especially notable in light of two features of the present study: first, the specific network model investigated in this analysis consisted of regions associated with DMN, a network thought to be active during rest. Second, the nodes of the DCM were located around peak coordinates of a rs-fMRI component, where only the choice of the specific component was guided by the ToM activation map, hence the analysis was more biased in favour of the RS condition. The power spectra in Figure 6 further emphasize the possible confounding by physiological variables when investigating connectivity in rs-fMRI data. Despite the notably higher amplitudes in the neural power spectrum of the t-fMRI data, differences in the observed BOLD power spectrum between rs- and t-fMRI are almost indiscernible.

The lower information gain observed with rs-fMRI data may also partly be attributable to the effect of noise correction based on signal from white matter and cerebrospinal fluid. As mentioned earlier, physiological noise may occupy the same frequency bands as the low-frequency RS activity (Liu, 2016), and regressing them out from the signal may also remove some of the signal of interest. A recent study demonstrated that global noise regression reduced information gain but increased information certainty over effective connectivity parameters (Almgren et al., 2020). It is in accordance with the higher information gain connected to t-fMRI in our study, however, with the difference that global signal regression is a more radical noise correction method than the one applied in the present analysis. It also accords with the slightly higher certainty connected to rs-fMRI, which again is explicable by the decreased within and between variance observed with such noise correction methods (Birn et al., 2014). Physiological noise and noise correction are fundamental issues in fMRI data analysis, and the effect of different methods of noise correction on both t-fMRI and rs-fMRI data can be further investigated with the present information-theoretic approach.

The final quality index, that of model discrimination ability, was low for both datasets, and information gain over model structure was not significantly different across the datasets. This statistic quantifies how readily similar models can be distinguished, where the models differ only in whether particular connections are switched on or off (by setting permissive or restrictive priors on those connections respectively). One likely explanation is that switching on or off individual connections in the model made only a small difference in the model evidence (free energy), due to covariance among the parameters. This may have been compounded by the short length of the time series subjected to DCM analysis, relative to the number of parameters in the model. Thus, finding sufficiently strong evidence for switching off any individual connection would be difficult.

It is important to note that alternative approaches to group-level analysis of rs-fMRI data may result in different peak coordinates when specifying nodes for DCM. The influence of such data analysis choices on information value is an important topic to cover in future studies. Furthermore, due to our interest in quantifying information value in the context of a network underlying ToM processes, we selected the rs-fMRI component that most closely matched activation patterns during the ToM condition. This means that there may have existed other partly overlapping rs-fMRI components with higher information value. Thus, our conclusions pertain only to this particular network, and not the informative value of rs-fMRI in general. More general statements about rs-fMRI will require extensive work that covers different cognitive systems and networks, as well as several alternative tasks. When considering the clinical utility of the imaging protocol, suitability of the task for the given patient population must be considered and similar analyses repeated on patient data. Furthermore, the superiority of the task fMRI data was here demonstrated with csd-DCM, and the results cannot be directly generalized to other models or analysis methods.

## CONCLUSION

The main finding of the present study is that a social cognition task (ToM) gave rise to more informative inferences about the effective connectivity of regions of the DMN than was enabled by rs-fMRI. The ToM task elicited stronger connections among regions compared to rs-fMRI, causing an increase in the measured *information gain*. The brain regions we examined were identified based on their activation during rs-fMRI, thus even for brain regions typically associated with activation during rest, there is a compelling argument for using an experimentally controlled task such as ToM to investigate them. Higher information gain due to stronger effective connectivity is an advantage in situations where the detectability of a network and reliability of the connectivity parameters are crucial, such as in clinical contexts. The present results therefore speak for an active task condition, such as the ToM task, to be preferred over rs-fMRI when investigating the (dys)function of the associated neural system. The strength of DCM is that it discriminates between neural and physiological sources of signal. The present results are therefore of interest also from a theoretical point of view, with the much weaker connectivity observed during rest supporting the assumption that the resting functional connectivity of DMN to a large part reflects physiological rather than neural processes.

With this study we wanted to demonstrate the utility of the BDC framework for the present issue and it should be considered a starting point for such analyses. The present investigation may be extended to neural networks underlying other cognitive and affective domains according to current theoretical and clinical research questions where the development of a suitable imaging paradigm is of interest.

## ACKNOWLEDGMENTS

Data were provided by the Human Connectome Project, WU-Minn Consortium (Principal Investigators: David Van Essen and Kamil Ugurbil; 1U54MH091657) funded by the 16 National Institutes of Health and Centers that support the NIH Blueprint for Neuroscience Research; and by the McDonnell Center for Systems Neuroscience at Washington University.

## SUPPORTING INFORMATION

Supporting information for this article is available at https://doi.org/10.1162/netn_a_00302. All data were obtained from the Human Connectome Project database (ConnectomeDB) and are available to investigators upon application. Analysis scripts used here are available from https://github.com/picusacademicus/RSvsToM.

## AUTHOR CONTRIBUTIONS

Julia Axiina Tuominen: Conceptualization; Formal analysis; Investigation; Methodology; Supervision; Validation; Visualization; Writing – original draft; Writing – review & editing. Karsten Specht: Conceptualization; Formal analysis; Funding acquisition; Investigation; Methodology; Project administration; Supervision; Validation; Visualization; Writing – review & editing. Liucija Vaisvilaite: Conceptualization; Investigation; Supervision; Writing – review & editing. Peter Zeidman: Conceptualization; Methodology; Software; Validation; Writing – review & editing.

## FUNDING INFORMATION

Karsten Specht, Norges Forskningsråd (https://dx.doi.org/10.13039/501100005416), Award ID: 276044.

## TECHNICAL TERMS

Effective connectivity: Causal influence directed from one brain region to another.
Intrinsic functional connectivity: Consistent temporospatial correlations among brain regions that emerge in the absence of experimental perturbation.
Entropy: Amount of disorder or randomness in a system, or in statistical terms, lack of information associated with probability distributions.
Bayesian data comparison (BDC): Method to systematically compare the quality of two or more datasets based on information theory.
Dynamic causal modelling (DCM): A generative model of effective connectivity that incorporates information on the hemodynamic response and a model of unobservable neural dynamics.
Theory-of-mind (ToM): Mental function involving social-cognitive and self-referential processes.
Default mode network (DMN): Medial fronto-parietal network that deactivates to a range of cognitive tasks and activates during rest and to tasks requiring self-referential processing.
DCM for cross-spectral densities (csd-DCM): Estimates effective connectivity from correlations in amplitudes and phase delays of fMRI time series, applicable to both resting-state and task fMRI data.
Parameter certainty: Encodes variance and covariance among parameters, defined as negative entropy of the covariance matrix.
Information gain: Relative entropy, that is, reduction in uncertainty after seeing the data relative to the uncertainty before seeing the data.
