# Supplementary material for: An information-theoretic analysis of resting-state versus task fMRI
Source: Netw Neurosci. 2023 Jun 30;7(2):769–86. doi: 10.1162/netn_a_00302 (PMC10312267; doi:10.1162/netn_a_00302)
Supplement: Supplementary file 1 [file netn-7-2-769-s001.pdf]

Tuominen, J., Specht, K., Vaisvilaite, L. & Zeidman, P. (2023). Supporting information for "An information-theoretic Analysis of resting-state versus task fMRI." *Network Neuroscience*. Advance publication. https://doi.org/10.1162/netn_a_00302

Supplementary material

Table S1

Group-level activation peaks during the ToM task with the contrast Social > Random

MNI[1]				
x	y	z	Cluster size	T-value
54	-52	16	1636	10.97
-54	-58	12	743	9.82
52	30	4	974	9.77
-28	-96	-4	169	9.30
8	12	64	88	8.94
-24	-76	-36	377	8.73
50	6	-22	180	8.06
48	-24	-6	157	8.01
-46	14	22	10	5.84
42	-38	40	3	5.79
36	-38	-24	2	5.79
-26	-46	-14	5	5.59
42	-76	26	1	5.44

[1]Montreal Neurological Institute brain coordinates

Note: The table includes whole-brain coordinates using the FWE corrected threshold of $p < 0.05$ with no restrictions to the number of voxels per cluster.

Formula S1

$$D_{KL}(P \parallel P_0) = \sum_{j=1 \dots k} (P_j \, ln \; P_j) + \ln k$$

For a single dataset, information gain over models is defined as KL-divergence between the posterior and prior model probabilities, where k = the number of models.

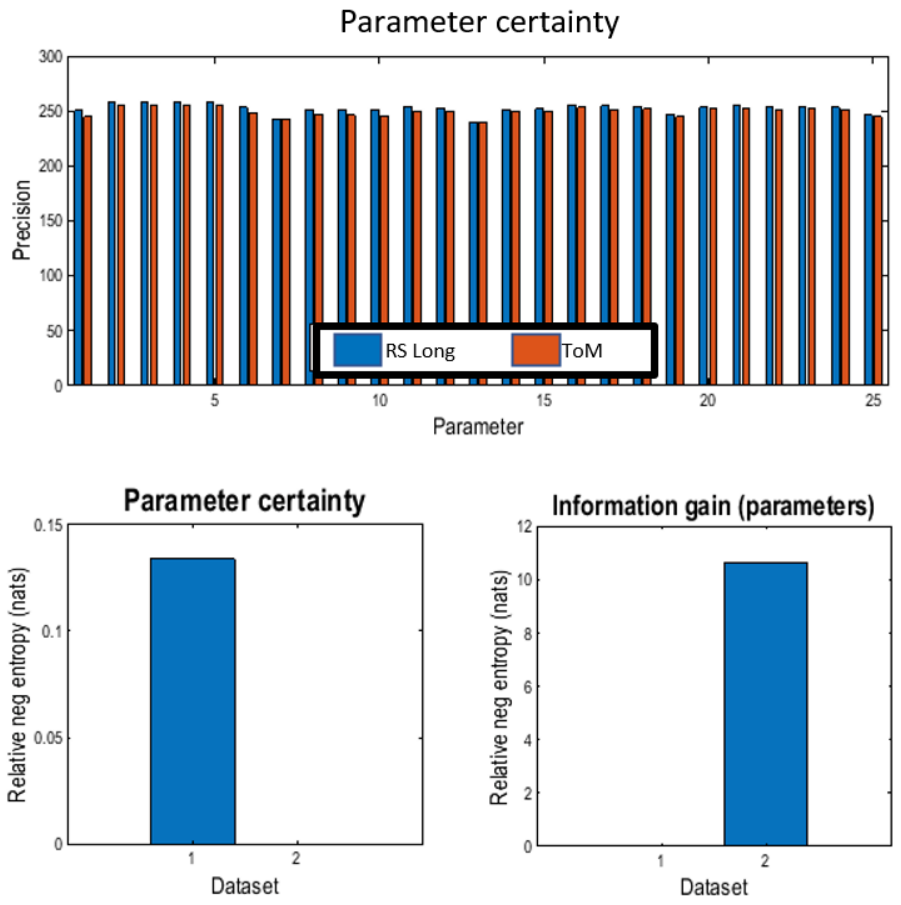

Figure S1. Parameter certainty and information gain over parameters in units of nats. Dataset 1 stands for full-length RS and dataset 2 for ToM. Parameter certainty and information gain over parameters are negative entropies in units of nats, which are presented relative to the worst performing dataset (by subtracting the negative entropy of the best performing dataset from that of the worst performing dataset).
